# You can’t always get as much iron as you want: how rice plants deal with excess of an essential nutrient

**DOI:** 10.3389/fpls.2024.1381856

**Published:** 2024-07-19

**Authors:** Andriele Wairich, May Sann Aung, Felipe Klein Ricachenevsky, Hiroshi Masuda

**Affiliations:** ^1^ Department of Agronomy and Crop Physiology, Justus Liebig University Giessen, Giessen, Germany; ^2^ Department of Biological Production, Faculty of Bioresource Sciences, Akita Prefectural University, Akita, Japan; ^3^ Botany Department, Institute of Biosciences, Federal University of Rio Grande do Sul, Porto Alegre, RS, Brazil; ^4^ Center of Biotechnology, Federal University of Rio Grande do Sul, Porto Alegre, RS, Brazil

**Keywords:** iron excess, rice, iron toxicity, reactive oxygen species, nutrient

## Abstract

Iron (Fe) is an essential nutrient for almost all organisms. However, free Fe within cells can lead to damage to macromolecules and oxidative stress, making Fe concentrations tightly controlled. In plants, Fe deficiency is a common problem, especially in well-aerated, calcareous soils. Rice (*Oryza sativa* L.) is commonly cultivated in waterlogged soils, which are hypoxic and can cause Fe reduction from Fe^3+^ to Fe^2+^, especially in low pH acidic soils, leading to high Fe availability and accumulation. Therefore, Fe excess decreases rice growth and productivity. Despite the widespread occurrence of Fe excess toxicity, we still know little about the genetic basis of how rice plants respond to Fe overload and what genes are involved in variation when comparing genotypes with different tolerance levels. Here, we review the current knowledge about physiological and molecular data on Fe excess in rice, providing a comprehensive summary of the field.

## 1 Introduction

Iron (Fe) is a transition metal that is essential for almost all living organisms. In plants, Fe is required for plant growth and development (Marschner, 1995). Because of its redox-active nature, Fe plays a crucial role in metabolic processes such as photosynthesis, respiration, nitrogen fixation, and assimilation (Evans and Russel, 1971; Balk and Schaedler, 2014; Connorton et al., 2017). Although Fe is the fourth most abundant element on the Earth’s crust, accounting for over 5% of the mass, Fe is usually found as low solubility ferric (Fe^3+^) oxides in the soil, which limits Fe supply for plant uptake. The problem is particularly common in calcareous soils, which cover approximately one-third of the Earth’s surface, making plants prone to Fe deficiency. The main symptoms associated with Fe deficiency are chlorosis, reducing the biomass, yield, and nutritional value of grains (Guerinot and Yi, 1994). Therefore, Fe deficiency is a problem for agricultural crops.

Fe uptake strategies in plants were characterized years ago, in which reduction strategy (or Strategy I) was associated with non-graminaceous plants and chelation strategy (or Strategy II) was linked to graminaceous (Poaceae family) species (Kobayashi and Nishizawa, 2012; Brumbarova et al., 2015). Strategy I relies on lowering soil pH through the extrusion of H^+^ ions to enhance the solubility of Fe^3+^, reducing Fe^3+^ to ferrous (Fe^2+^) at the root surface and absorption of Fe^2+^ into root cells by the Fe high-affinity transporter IRT1 (iron-regulated transporter) (Eide et al., 1996; Robinson et al., 1999; Santi and Schmidt, 2009). In contrast, Strategy II relies on the release of phytosiderophores (PS) into the rhizosphere, which forms a complex, Fe(III)–PS, which is transported into root cells through specific transmembrane proteins of the Yellow Stripe (YS) family (Inoue et al., 2009). Recent work on the secretion of phenolic compounds upon Fe deficiency and transcriptional regulation of Fe uptake has blurred this clear division, suggesting that these groups may share aspects of Fe uptake mechanisms (Grillet and Schmidt, 2019). Furthermore, work on wild rice showed that the combined strategy described in cultivated Asian rice, including a full chelation strategy as found in other Poaceae species (Wairich et al., 2019) as well as the transporter involved in the reduction strategy (Ishimaru et al., 2006), is shared by closely related wild *Oryza* species.

In contrast to Fe deficiency, Fe excess tolerance mechanisms are not well understood. In anaerobic or waterlogged conditions, ferric Fe is reduced to ferrous (Fe^2+^) ions, which are more soluble and may be excessively taken up by roots. In this scenario, Fe can be cytotoxic, disrupting several plant metabolic and physiological processes such as carbon metabolism, respiration, photosynthetic efficiency, and enzyme activities. When it exists as a free form in the cytosol, excess Fe^2+^ can cause the toxic reaction with •OH by the Fenton reaction (Fenton, 1894), leading to cellular toxicity (Becker and Asch, 2005; Sperotto et al., 2012; Wairich et al., 2021), and causes oxidative stress, mainly due to reactive oxygen species (ROS) formation since Fe participates in Fenton chemistry, which leads to overproduction of ROS, particularly hydroxyl radical •OH (Becana et al., 1998).

Rice is one of the most important cultivated cereals in the world, and it is widely cultivated in lowland, flooded areas, which can lead to Fe excess. Fe toxicity is one of the most serious abiotic stresses in wetland cultivation conditions in Asia, Africa, and South America (Audebert and Sahrawat, 2000) and can reduce yield from 12% to 100% depending on the Fe toxicity intensity and cultivar tolerance (Sahrawat, 2004). Fe also precipitates in the root apoplast, forming a typical orange/brown Fe plaque, which can decrease uptake or other essential nutrients, further hampering plant growth and development (Sahrawat, 2004). Therefore, rice plants need to tightly control Fe uptake and concentration to avoid excess while providing sufficient Fe for normal physiological functions.

Generally, it is proposed that Fe toxicity tolerance can be achieved by (Defense I) Fe exclusion by roots, which involves Fe exclusion from entering root cells by the formation of the Fe plaque in the apoplast, consisting mostly of precipitated Fe (Deng et al., 2010), (Defense II) Fe retention in roots, and suppression of Fe translocation to shoots by manipulating root apoplastic barriers such as lignification (Stein et al., 2019), or by restricting root-to-shoot Fe movement (Wairich et al., 2021); (Defense III) Fe compartmentalization in shoots, based on the Fe compartmentalization in organelles such as the vacuoles or Fe storing proteins such as ferritins (Stein et al., 2009); or (Defense IV) detoxification of ROS, which rely on increasing protection against the deleterious effects of Fe overload, such as accumulation of antioxidants (Wu et al., 2014; Wu et al., 2017). In addition to the proposed mechanisms and the eventual identification of genotypes that tolerate Fe overload based on them, the key genes involved in such mechanisms are poorly characterized. Here, we summarize the current knowledge on how rice plants respond to Fe excess and which genes have their function related to Fe excess tolerance.

## 2 Fe dynamics in soil and rhizosphere

Fe solubility is a critical factor, often even more than Fe content in the soils. Also, water condition (soil redox state) significantly affects the Fe solubility in the soil. Since rice is commonly grown under flooded conditions, rice plants are easily exposed to Fe toxicity. Soils with good aeration typically contain elevated levels of ferric iron (Fe^3+^), which has limited solubility. However, waterlogging creates an oxygen-deprived environment that converts Fe^3+^ to the more soluble ferrous iron (Fe^2+^), leading to high solubilities of Fe in the soil solution and higher uptake by rice roots (Becker and Asch, 2005). Soluble Fe concentrations are approximately 50–100 ppm in flooded soil but approximately 0.1 ppm in non-flooded soil (Ponnamperuma, 1978). Waterlogged soils, particularly on a range of soil types including fluvisols, gleysols, podzols, acrisols, and ferralsols, may lead to Fe toxicity (Chérif et al., 2009; Yang et al., 2018). Poor drainage or poor water management leads to the deterioration of soil properties and promotes Fe^2+^ increased concentration in the soil solution; thereby, Fe^2+^ is readily absorbed by plants, causing Fe toxicity (Audebert and Fofana, 2009).

**Figure 1:**
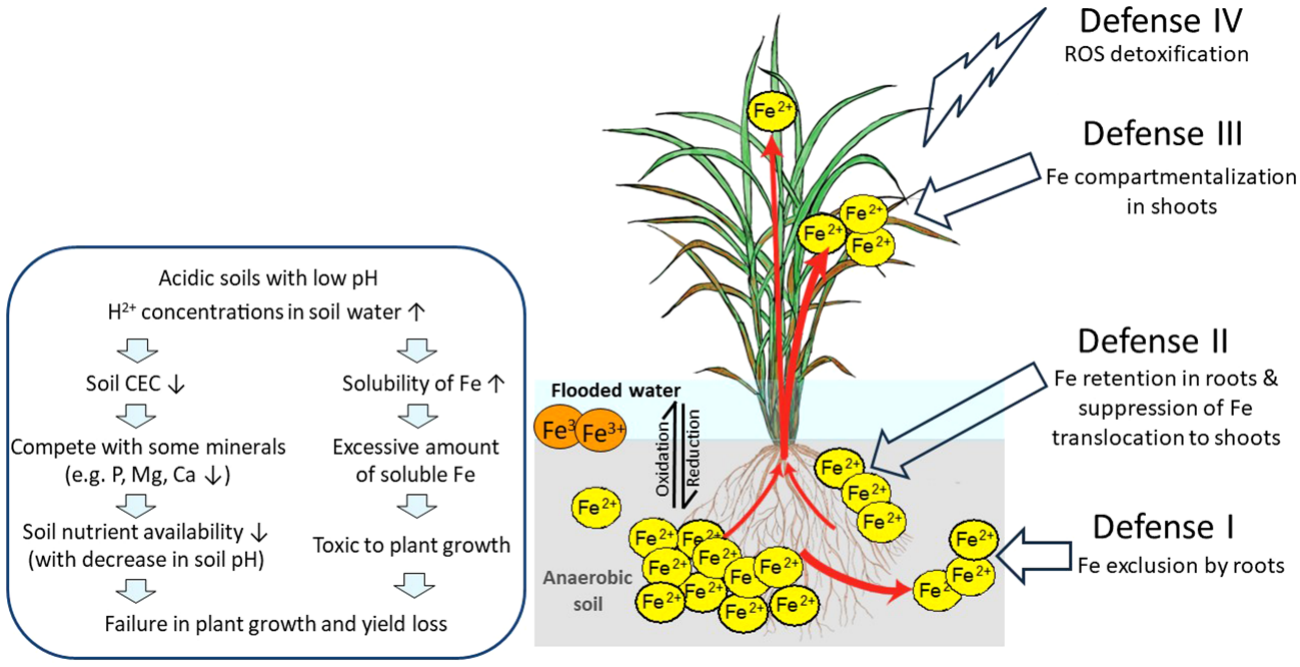
Effects of Fe toxicity on rice plants in flooded low pH soils and possible defense mechanisms of rice against excess Fe. In flooded soils with anaerobic and low pH conditions, ferric ion (Fe^3+^) is reduced to the more soluble ferrous ion (Fe^2+^), followed by loss of rice yield. Fe^3+^, sparingly soluble ferric ion; Fe^2+^, soluble ferrous ion; ROS, reactive oxygen species; red arrows, Fe uptake and transport by rice; DC, discrimination center. This figure is modified from Aung and Masuda (2020).

The soil pH greatly influences Fe solubility in soil solution. Plant Fe deficiency occurs due to low Fe availability in aerobic soils particularly in high pH and/or calcareous soils since the increase of the pH by one unit decreases by 1,000-fold the availability of Fe due to the formation of insoluble Fe(III)-hydroxide. In contrast, plant Fe toxicity mainly occurs because of high Fe availability in anaerobic soils and high Fe soluble in low pH acidic soils (**Figure 1**). In such types of soils, an increase in H^+^ concentrations in soil solution leads to a decrease in cation exchange capacity (CEC). In addition to naturally acidic soils, intensive farming and overuse of nitrogen (N) fertilizer and industrial activities contribute to soil acidification. Low pH elevates the solubility of heavy metal elements, such as Fe, copper (Cu), manganese (Mn), zinc (Zn), and aluminum (Al) (Bian et al., 2013), and decreases the plants’ absorption of other minerals such as phosphorus (P), magnesium (Mg), and calcium (Ca) due to competition of excessive H^+^ ions in low pH (Poschenrieder et al., 1995).

## 3 Morpho-physiological effects of Fe toxicity on plant growth

Fe toxicity disrupts several morpho-physiological processes. Symptoms of Fe excess toxicity are identifiable in both roots and leaves. The initial visual symptom of plant growth under Fe excess is the reduction or stunting in root growth. Treatments with different levels of Fe excess [×10, ×20, ×30, ×50, ×70, ×100, and ×150 of Fe excess compared to ×1 of Fe (35.7µM)] under acid pH (4.0) during rice seedling stages revealed that root growth inhibition was strongly associated with the severity of Fe excess toxicity (Aung et al., 2018b). Root tips contact external Fe, which impairs primary root growth by decreasing cell division and elongation as well as the growth of lateral roots (Zhang et al., 2012a; Li et al., 2015). Moreover, the higher the Fe excess levels, the lower the other nutrients in roots, including Zn, Cu, Mn, and Ca (Aung et al., 2018b). The symptom occurrences on old leaves are more severe at higher Fe concentrations, and when Fe excess reaches ×100 or ×150 the concentration of the control condition, even new leaves are strongly affected by Fe toxicity (Aung et al., 2018b). Excess Fe also limits roots’ ability to scavenge for nutrients, causing a poorly developed root system, secondary nutrient deficiencies in plants, Fe overload in shoots and significant reduction in leaf development, and lowered grain yields.

Massive amounts of Fe are transported from roots to shoots via the xylem by transpiration stream, causing cellular Fe overload and damage in plant aerial tissues (Briat and Lobreaux, 1997). Reddish brown spots or bronzing on leaves are visible symptoms of Fe excess in plants. The bronzing symptom on leaves is due to the accumulation of oxidized polyphenols (Peng and Yamauchi, 1993). Leaf bronzing is strongly related to yield loss. Leaf bronzing symptoms are visually determined on a scale of 10 levels, ranging from 0 (no symptoms) to 10 (complete damage with leaves dead) (IRRI, 1996). A study correlating yield data and leaf bronzing symptom formation showed that a unit increase in bronzing score symptoms resulted in approximately 400 kg/ha yield loss (Audebert and Fofana, 2009). Although root growth has stopped, the shoot still can grow under Fe excess and maintain other minerals such as Zn, Cu, and Ca in leaves similar to control plants (Aung et al., 2018b). Interestingly, the greater the Fe excess, the more Fe was translocated preferentially to the old or mature leaves compared to young leaves (Aung et al., 2018b), suggesting a mechanism to partition excess Fe to the mature leaves to avoid damage to new leaves.

Fe can exist in cells as Fe^2+^ and Fe^3+^ ions. Recently, a regulated cell death named ferroptosis was identified in mammals and later shown to occur in plants (Distéfano et al., 2017; Dangol et al., 2019). Ferroptosis is an Fe-dependent regulated cell death: Fe causes ROS accumulation, which leads to peroxidation of membrane-localized phospholipid containing polyunsaturated fatty acids and glutathione and ascorbic acid depletion (Distéfano et al., 2017). Interestingly, ferroptosis was linked to heat stress in *Arabidopsis thaliana* (Distéfano et al., 2017) and to hypersensitive response upon *Magnaporthe oryzae* infection in rice (Dangol et al., 2019). Another report suggested that Fe supplementation increases ferroptotic cell death when plants are infected with a virulent tobacco mosaic virus (Macharia et al., 2020). However, it is not known whether ferroptosis is involved in Fe excess-caused morphological and anatomical changes in rice plants and whether proteins involved in ferroptotic cell death may regulate tolerance to excessive Fe.

## 4 Plant defense mechanisms to Fe excess toxicity

In general, rice plants employ four defense mechanisms: Defense I (Fe exclusion by roots), Defense II (Fe retention in roots and suppression of Fe translocation to shoots), Defense III (Fe compartmentalization in shoots), and Defense IV (detoxification of ROS) (Aung and Masuda, 2020, Aung and Masuda, 2023). We have proposed the model shown in **Figure 1**.

### 4.1 Defense I: Fe exclusion by roots

When Fe excess occurs, rice plants apply Defense I mechanism where the root system prevents excessive Fe uptake. Fe excess-tolerant rice genotypes that use these mechanisms possess high Fe exclusion ability by roots. These genotypes employ the oxygen release or enzymatic oxidation for the oxidization and precipitation of Fe^2+^ to Fe^3+^, on the root surface, and avoid excess Fe^2+^ from uptake into rice shoots. The precipitation of Fe on the root surface forms the Fe plaque, which acts as a physical barrier to further uptake of Fe^2+^ ions into root tissues (Becker and Asch, 2005). Mahender et al. (2019) reported that tolerant genotypes with extensive Fe plaque have high Fe exclusion ability. In addition, high root oxidation in Fe-exclusion genotypes had wider pith cavity diameters in shoots, increased root aerenchyma volume, and greater numbers of lateral roots, all favoring internal oxygen movement (Wu et al., 2014). Furthermore, silicon application has a dual benefit: directly reducing Fe uptake in rice (Ma and Takahashi, 1990) and strengthening Casparian bands in the root endodermis, which acts as a diffusion barrier to prevent excessive Fe uptake (Hinrichs et al., 2017; Becker et al., 2020), therefore alleviating Fe excess toxicity. Moreover, high lignification in the roots can enhance Fe overload tolerance, as increased lignification was found in the cortex and the vascular bundle of one tolerant genotype in comparison to a susceptible genotype (Stein et al., 2019). Therefore, Fe exclusion can be based on changing Fe access to root symplast entry points. However, the molecular regulators of such mechanisms are not known.

### 4.2 Defense II: Fe retention in roots and suppression of Fe translocation to shoots

Though under Fe excess conditions, plants need to absorb other essential metals such as Zn, Cu, and Mn. Therefore, the metal transporters in roots take up Fe while absorbing other essential metals (Aung and Masuda, 2020). Thus, Defense I Fe exclusion may be insufficient (Aung and Masuda, 2020). In such conditions, plants may employ Defense II, retaining excess Fe in roots and preventing its translocation to shoots (Tadano, 1975), thereby limiting Fe accumulation in leaves (Becker and Asch, 2005; Silveira et al., 2007). When the tolerant genotype EPAGRI 108 was grown in the field with Fe toxicity (pH ranging from 4.8 to 5.2 and Fe concentration in the soil solution reaching 284 mg/L), a higher Fe concentration was observed in root symplast cells (7 mg/g dry weight) than that of the sensitive genotype BR IRGA 409 (1.93 mg/g dry weight) (Stein et al., 2014). They reported that approximately 94% of the Fe was allocated in root apoplasts and the lower Fe concentration in the leaves of the tolerant genotypes, showing the inhibition of root-to-shoot Fe translocation and the employment of Defense II mechanisms. Moreover, protecting new leaves from Fe overload is important for the survival of the plants. The region named discrimination center (DC) located at the basal part of the shoot of graminaceous plants is where metals absorbed by roots primarily accumulate and is an important tissue regulating Fe partitioning to the aerial parts of plants that may also act as a barrier for root-to-shoot transport of excessive Fe (Aung and Masuda, 2020).

### 4.3 Defense III: Fe compartmentalization in shoots

When the Defense II mechanism is inadequate to sequester excess Fe in root tissues, plants may transport Fe from roots to shoots (Aung and Masuda, 2020). When Fe over-accumulates in shoots, plants employ Defense III, which consists of chelation, isolation, or sequestration of excess Fe to mature leaves or other less photosynthetically relevant tissues to store Fe in a safe form. Better shoot growth and a higher shoot Fe concentration were found in some tolerant rice genotypes (Onaga et al., 2013). For example, rice genotypes, namely, Siam Saba, Mahsuri, Margasari, and Pokkalli, have shown lower leaf bronzing, higher grain yields, and higher shoot Fe concentrations, representing shoot-based tolerance (Nugraha et al., 2016). When plants were grown in hydroponic culture, shoot Fe concentrations proportionately increased with an increase in Fe excess levels in culture (Aung et al., 2018b). However, a ninefold increase in root Fe concentrations was observed under 20 times Fe excess treatment; thereafter, root Fe concentrations became constant throughout in higher Fe treatment levels. Therefore, the capacity to retain excess Fe in root tissues is often limited in rice plants. Once Fe levels are enhanced and cannot be avoided by Defense II, plants translocate excess Fe from roots to shoots. Old or mature leaves usually show bronzing symptoms earlier than new leaves, demonstrating earlier and greater Fe translocation to old leaves in association with Fe overload levels in plants. Thus, through the regulation function of the DC, Fe is preferentially transported and allocated to old leaves rather than new leaves, avoiding Fe excess damage in new leaves (Aung et al., 2018b; Aung and Masuda, 2023).

### 4.4 Defense IV: detoxification of ROS

In plants, abiotic stresses like salinity and Fe toxicity often produce ROS including O_2_^−^, OH^−^, and H_2_O_2_ (Galaris et al., 2019). In plant tissues, Fe^2+^ ions exist in a highly soluble and reactive form. Fe excess may prevent free divalent Fe from being sequestered safely, causing excessive Fe accumulation in the cytosol and toxic reactions such as the Fenton reaction (Aung and Masuda, 2020). The Fenton reaction induces ROS overproduction particularly the cytotoxic hydroxyl radical (•OH) (Becana et al., 1998; Thongbai and Goodman, 2000). The ROS production in plants leads to oxidative damage to plant membranes (Miller et al., 2008) and subsequent programmed cell death (de Pinto et al., 2012). In particular, Fe overload in chloroplasts causes ROS-driven oxidative damage (Balk and Schaedler, 2014). Cellular oxidative damage caused by Fe excess leads to several changes in morpho-physiological and yield traits of rice (Hell and Stephan, 2003; Sikirou et al., 2015).

However, plants employ a defense mechanism categorized as Defense IV, detoxification of ROS. Plants usually cope with oxidative stress caused by any abiotic stress/ROS damage by a unique antioxidative defense system that includes various antioxidant enzymes like superoxide dismutase (SOD), catalase (CAT), peroxidase (POD) (Bode et al., 1995; Fang and Kao, 2000), and also non-enzymatic antioxidants like ascorbate and glutathione (Fang et al., 2001; Gallie, 2013). In addition, comprehensive microarray analyses of the rice grown with different Fe excess intensities revealed that the genes contribute to Defense IV work at the molecular level in severe Fe excess conditions, which causes a significant reduction in plant growth (Aung et al., 2018b). For example, superoxide dismutase (Os08g0561700) was induced two to three times in Fe excess newest leaves; peroxidase (Os07g0531400) was induced 30–100 times in Fe excess roots; haem peroxidase family protein (Os01g0327100) induced 20–150 times in Fe excess newest and old leaves (Aung et al., 2018b). Thus, these genes involved in ROS metabolism may play an important role in Fe excess defense. Furthermore, plants also induce the expression of genes related to oxidative stress, oxygen, and electron transfer and those encoding cytochrome P450 family proteins in response to severe Fe excess (Quinet et al., 2012; Aung et al., 2018b). Moreover, transcription factors that are related to cell death and stress responses (*OsNAC4*, *OsNAC5*, and *OsNAC6*) were also highly induced in response to Fe excess (Finatto et al., 2015; Aung et al., 2018b). These ROS-related genes may be critical to mitigate Fe excess stress and tolerant mechanisms in plants.

## 5 Possible approaches to overcome iron excess toxicity

Some agronomic practices to cope with Fe toxicity have been reported. Lime addition to soil has been the most common practice for increasing soil pH for several years. The lime application increases root cell growth, lowers absorption of Fe, and enhances the protective ability of the cells (Bian et al., 2013). Silicon application has also been reported to alleviate Fe toxicity (Ma and Takahashi, 1990). Magnesium application was recently shown to reduce Fe toxicity (Rajonandraina et al., 2023). Application of sodium bentonite (NaB) improved highly acid sulfate soils from low pH (~3.5) to normal pH (~6.5), reducing aluminum and Fe toxicity in soils. The extractable Fe concentration was remarkably reduced in the plots after applications of NaB by enhancing higher pH conditions (Tawornpruek et al., 2023). Moreover, field drainage practices may mitigate Fe toxicity. Two cycles of water suppression or drainage in the field result in higher grain yields in both sensitive and resistant rice cultivars than continuous irrigated control field (de Campos Carmona et al., 2021).

Fe excess problem occurs because low pH soil causes Fe^2+^ ions to be readily solubilized. Lime application is one of the efficient agricultural methods to increase soil pH. However, a large amount of lime is required, and it needs to be applied almost every year to maintain soil pH at a low level. Thus, this practice may be difficult for farmers. Once Fe excess-tolerant rice varieties are produced by breeding, it would be cost-efficient and sustainable to overcome the Fe excess problem in rice cultivation. Although there are also several other approaches, they are costly and ecologically unsound. Furthermore, Fe uptake, Fe translocation, and Fe allocation must be strictly regulated in plants (Kobayashi and Nishizawa, 2012). Plants vary significantly in Fe excess tolerance (Nugraha et al., 2016; Sikirou et al., 2016; Kar et al., 2021). This diversity or variation in acid soil tolerance makes it possible to breed tolerant cultivars. The success of breeding programs depends on an understanding of the physiology, genetics, and gene regulation mechanism of Fe toxicity tolerance. There were works that screened Fe excess-tolerant varieties or produced Fe excess tolerance by breeding approaches (Sikirou et al., 2016; Kar et al., 2021). Therefore, breeding and use of tolerant rice cultivars are a possible alternative and the most efficient way for rice production on Fe-toxic soils.

## 6 Transcriptional responses of rice plants exposed to iron excess

Several studies have analyzed transcriptional responses in root and/or shoots to Fe excess using different Fe sources, Fe concentrations, treatment duration, and analysis methods. We summarize these studies in **Table 1** to facilitate comparisons between studies. Despite the large differences in experimental design, some commonalities were observed (**Table 1**). For example, FeSO_4_ is the main source of Fe used for Fe excess treatments, with FeCl_2_ and Fe-EDTA as other sources used. There are studies covering a wide range of concentrations and treatment times, and the threshold toxic concentration is likely to vary depending on the experimental system, but concentrations above 4.47 mM are used in shorter treatments (up to 7 days), while lower concentrations such as 2.5 mM allow longer treatments (14 days; **Table 1**).

**Table 1:** Comparison of different transcriptomic studies of Fe excess responses in rice (*Oryza sativa* L.).

Fe source	Concentration^a^	Duration of stress	Genotype	Tissue	References
FeSO_4_	(2.24 mM) 125 mg/L	3 days, 3 weeks	*O. sativa*, cv. ‘I Kong Pao’ (IKP)—tolerant to Fe toxicity	Shoot, root	(Quinet et al., 2012)
FeSO_4_	(4.47 mM) 250 mg/L	7 days	*O. sativa*, cv. Curinga *Oryza meridionalis* Introgression line (*O. sativa × O. meridionalis*)	Shoot	(Wairich et al., 2021)
FeSO_4_	(8.95 mM) 500 mg/L	3 days	*O. sativa*, cv. BR-IRGA 409 (sensitive) *O. sativa*, EPAGRI 108 (tolerant)	Root	(Stein et al., 2019)
FeSO_4_	15 mM	2 days	*O. sativa* Lachit (tolerant) *O. sativa* Hacha (sensitive)	Shoot, root	(Kar et al., 2021)
FeSO_4_	(17.9 mM) 1,000 mg/L	4 days	*O. sativa*, cv. IR29 (sensitive) *O. sativa*, FL483 (tolerant)	Shoot	(Wu et al., 2017)
FeCl_2_	0.36, 0.71, 1.79, 2.50 mM	14 days	*O. sativa* cv Tsukinohikari	Root, DC, stems, old and new leaves	(Aung et al., 2018b)
Fe^2+b^	(6.98 mM) 390 mg/L	18 days	*O. sativa*, cv. Nipponbare (sensitive)	Shoot	(Finatto et al., 2015)
Fe-EDTA	2.5 mM	3 days and 2 weeks	*O. sativa* ssp. *aromatic* Keteki Joha	Shoot, root	(Regon et al., 2022)
Fe-EDTA	0.5 mM^c^	1 week	*O. sativa*, cv. Nipponbare (sensitive to Fe toxicity)	Shoot, root	(Bashir et al., 2014)

Tolerant or sensitive means tolerant or sensitive to Fe toxicity.

DC, discrimination center.

a Concentrations are given as reported by authors and converted to molarity for comparison.

b Fe source for Fe excess treatments not provided.

c Fe excess at this concentration is typically not toxic to rice. We kept the paper in order to be comprehensive.

We also provide a list of Fe excess-regulated genes in these studies, which can be used as markers for the breeding of Fe excess-tolerant rice (**Table 2**). As expected, genes encoding proteins involved in Fe uptake and/or induced by Fe deficiency were downregulated in roots. These include the genes encoding enzymes from the PS synthesis pathway, such as *OsDMAS1* (deoxymugineic acid synthase), *OsNAAT1* (nicotianamine aminotransferase 1), *OsSAMS1* (*S*-adenosyl-l-methionine synthetase 1), and *NAS* (nicotianamine synthase) genes; the PS efflux transporter *OsTOM1*/*OsZIFL4* (Transporter of Mugineic acid family/ZINC INDUCED FACILITATOR-like 4) (Nozoye et al., 2011; Ricachenevsky et al., 2011); the Fe(III)–PS complex uptake transporter *OsYSL15* (YELLOW STRIPE-LIKE 15) (Inoue et al., 2009; Lee et al., 2009), Fe(II) NA transporter *OsYSL2* (YELLOW STRIPE-LIKE 2) (Koike et al., 2004), and *OsYSL16* (YELLOW STRIPE-LIKE 16), which play a role in the allocation of Fe(III)-DMA via the vascular bundles (Kakei et al., 2012). Given that PS is synthesized, effluxed, and acquired by rice roots under Fe deficiency, it is clear that the chelation strategy is shut down upon Fe excess. Conversely, *OsFER1* (Ferritin 1) and *OsFER2* (Ferritin 2) genes, encoding the two ferritin proteins in the rice genome, suggested to buffer rice plants against Fe overload (Stein et al., 2009), and the vacuolar Fe transporter 2 (*OsVIT2*) (Zhang et al., 2012b; Bashir et al., 2013; Che et al., 2021), which may protect cells against excessive Fe accumulation by vacuolar Fe detoxification (although its clear demonstration is still lacking—see section “Genes encoding proteins involved in Fe excess response in rice”) were upregulated in both roots and shoots (Quinet et al., 2012; Bashir et al., 2013, Bashir et al., 2014; Finatto et al., 2015; Aung et al., 2018b; Kar et al., 2021; Wairich et al., 2021). Genes characterizing an inclusion strategy such as *OsVIT2* and ferritin may play an important role in the detoxification of excess Fe in shoot tissues (Aung et al., 2018b). Taken together, these can be considered consistent markers for Fe excess in roots and shoots (**Table 2**). A few studies focused on identifying genes up- and downregulated by Fe excess, whereas others focused on comparing genotypes that contrast in tolerance/sensitivity. One early work showed that Fe excess induces very different transcriptomic responses when early (3 days) and late (3 weeks) stresses are compared, with early stress changing the expression of a larger gene subset (Quinet et al., 2012). Another one compared Fe excess and deficiency transcriptomic responses in both roots and shoots (Bashir et al., 2014), showing that few genes are oppositely regulated, while most of the responses seem unrelated. An analysis of Fe excess transcriptional responses in leaves suggested that long-term repeat retrotransposons play an important role in transcription response to Fe toxicity stress (Finatto et al., 2015). A more recent one analyzed Fe excess responses in an aromatic variety, proposing that ROS homeostasis is an essential defense mechanism under severe Fe^2+^ toxicity (Regon et al., 2022).

**Table 2:** Comparison of up- and downregulated marker genes across transcriptomic studies of Fe excess responses in rice (*Oryza sativa* L.).

Gene	Regulation	Quinet et al., 2012	Bashir et al., 2014	Finatto et al., 2015	Wairich et al., 2021	Stein et al., 2019	Regon et al., 2022	Wu et al., 2017	Aung et al., 2018b	Kar et al., 2021
*OsNAAT1* Os02g0306401	Downregulated		Root	Shoot ^1^	Shoot for *O. sativa* and *Oryza meridionalis* ^4^	Root for BR 409	Shoot ^3^		Root (all Fe levels tested) DC (0.36, 0.71 mM) Stem (2.50 mM) ^10^	Root
*OsNAS1* Os03g0307300	Downregulated	Root	Root			Root for BR 409	Root/shoot		Root, DC (all Fe levels) Stem (2.50 mM)	Root, shoot
*OsNAS2* Os03g0307200	Downregulated	Root	Root		Shoot for *O. meridionalis* ^4^	Root for BR 409	Root/shoot		Root, DC (all Fe levels) Newest leaf (2.50 mM) Old leaf (2.50 mM)^10^ 14	Root, shoot (except for shoots of Lachit)
*OsDMAS1* Os03g0237100	Downregulated		Root				Root		Root (all Fe levels) Stem (2.50 mM) ^10^	Root
*OsYSL2* Os02g0649900	Downregulated		Root		Shoot for *O. sativa* and *O. meridionalis* ^5^	Root for BR 409	Shoot		Root, DC (except DC 1.79 mM) Old leaf (all Fe levels, except 0.36 mM)	Root, shoot
*OsYSL15* Os02g0650300	Downregulated	Root	Root			Root for BR 409			Root (all Fe levels) DC (0.71 mM)	Root, shoot
*OsYSL16* Os04g0542800	Downregulated	Root			Shoot for *O. sativa* ^4^	Root for BR 409		Shoot	Root (2.50 mM) Stem (0.36 mM)	Root
*OsIRT1* Os03g0667500	Downregulated		Root			Root for BR 409 Root for EPAGRI 108 ^7^	Root		Root, DC (all Fe levels) Stem (1.79 mM)^10^ Old leaf (0.36, 0.71 mM)^10^	Root, shoot (except shoot of Lachit)
*OsIRT2* Os03g0667300	Downregulated		Root				Root/shoot		Root (all Fe levels) Stem (0.71, 1.79 mM) DC (0.71 mM)^10^	Root, Shoot of Hacha
*OsNRAMP1* Os07g0258400	Downregulated	Root	Root		Shoot for *O. sativa*	Root for BR 409	Root/shoot		Root (all Fe levels) Stem (0.36, 1.79 mM) DC (0.36 mM) Old leaf (0.71, 1.79, 2.50 mM)^10^	Root, Shoot
*OsTOM1* Os11g0134900	Downregulated		Root			Root for BR 409	Root		Root (all Fe levels) DC (0.71 mM)	Root
*OsIRO2* Os01g0952800	Downregulated		Root		Shoot for all genotypes		Root/shoot		Root (all Fe levels) DC (0.36, 0.71, 2.50 mM) Old leaf (1.79 mM), Newest leaf (0.36, 0.71 mM)	Root, shoot
*OsIRO3* Os03g0379300	Downregulated		Shoot		Shoot for *O. meridionalis*	Root for BR 409			Root (all Fe levels)	Root, Shoot
*OsMIR* Os12g0282000	Downregulated		Root			Root for BR 409	Root/shoot			
*OsFRO2* Os04g0578600	Downregulated		Shoot	Shoot^1^	Shoot for *O. sativa* and *O. meridionalis*		Shoot		DC and Stem	Shoot
*OsHRZ2* Os05g0551000	Downregulated		Shoot							Shoot of Hacha
*OsNAS3*	Upregulated					Root for BR 409	Root/shoots ^12^		Root (1.79 and 2.50 mM), DC (0.71 mM), old leaf (0.36, 0.71, 1.79, 2.50 mM), newest leaf (1.79, 2.50 mM)	Root/2 days Shoots (Hacha) ^13^
*OsSAMS1* Os05g0135700	Upregulated		Shoot							Shoot of Lachit
*OsNRAMP5* Os07g0257200	Upregulated	Root			Shoot for *O. sativa* and *O. meridionalis* ^6^		Root^2^/shoot^2^		DC (2.50 mM)^11^ Old leaf (1.79, 2.50 mM)^11^	Root, shoot ^9^ (except shoot of Lachit)
*OsVIT2* Os09g0396900	Upregulated			Shoot	Shoot for *O. sativa*	Root for BR 409			Root, DC, stems, old & new leaves (all Fe levels)	Root, Shoot
Vacuolar iron transporter homolog 2 Os04g0538400	Upregulated	Shoot and Root	Shoot	Shoot	Shoot for *O. sativa* and introgression line	Root (BR 409) Root for EPAGRI 108 ^8^	Shoot			Root and Shoot
*OsFER1* Os11g0106700	Upregulated	Root			Shoot for *O. sativa* and introgression line	Root for BR 409	Root/shoot		Root, old & new leaves (all Fe levels) DC (0.32, 1.79, 2.50 mM) Stem (2.50 mM)	Root, Shoot
*OsFER2* Os12g0106000	Upregulated	Root			Shoot for *O. sativa* and introgression line	Root for BR 409	Root/shoot	Shoot	Root, old & new leaves (all Fe levels) DC (0.32, 1.79, 2.50 mM) Stem (2.50 mM)	Root, Shoot

^1^ Finatto et al. (2015) identified these genes as upregulated.

^2^ Regon et al. (2022) identified these genes as downregulated.

^3^ Regon et al. (2022) identified these genes as upregulated.

^4^ Wairich et al. (2021) identified these genes as upregulated.

^5^ For *O. meridionalis*, the gene was upregulated.

^6^ For *O. sativa* and *O. meridionalis*, the gene was downregulated.

^7^ For *O. sativa* cv EPAGRI 108, the gene was upregulated.

^8^ For *O. sativa* cv EPAGRI 108, the gene was downregulated.

^9^ The gene was downregulated.

^10^ The gene was upregulated.

^11^ The gene was downregulated.

^12^ This gene was found as upregulated in roots but downregulated in shoots by Regon et al. (2022).

^13^ This gene was found as down in these tissues/genotypes by Kar et al. (2021).

Employing physiological and transcriptomic analysis to evaluate the response to five different levels of Fe excess in roots, DC, stems, old leaves, and new leaves, Aung et al. (2018b) identified that higher levels of Fe in the solution lead to higher accumulation of Fe in old leaves, a possible mechanism employed to avoid the damage caused by Fe toxicity to the new leaves. The authors proposed three zones of Fe excess levels: 1) the non-affected zone, 2) the affected zone, and 3) the dead zone. Considering each zone, the study showed that an exclusion strategy was observed for root tissue (Defense I) based on the suppression of genes involved in Fe uptake and transport. In the affected zone, a major change in the expression level of genes involved with the avoidance of ROS was observed. The authors suggested that plants may employ similar mechanisms in the non-affected and affected zones when considering the new leaves and roots; however, in old leaves, DC, and stem tissues, possibly a different mechanism is employed (Aung et al., 2018b).

Another set of studies explored transcriptional differences between genotypes with contrasting Fe tolerance in order to identify mechanisms and genes associated with higher capacity to withstand Fe excess (**Table 1**). In a pioneering study, two genotypes were physiologically and molecularly characterized with that intent (Wu et al., 2017). The authors hypothesized whether tolerance could be derived from 1) Fe uptake and storage, 2) antioxidant biosynthesis to scavenge ROS, and 3) increased enzymatic activity to counteract ROS and antioxidant turnover. Using transcriptional differences when comparing the two genotypes in both roots and shoots, authors found a shoot-based mechanism associated with the third hypothesis: Fe sensitivity was caused by *in planta* reduced ascorbate pro-oxidant activity together with Fe excess. In agreement with that, the tolerant genotype showed increased ascorbate oxidase and lower dehydroascorbate reductase activities. Interestingly, there was no difference in Fe accumulation in shoot tissues, showing that controlling Fe uptake and root-to-shoot translocation is not important for tolerance for this particular genotype. The work established an ascorbate redox state as a shoot-based Fe tolerance mechanism (Wu et al., 2017). This is an example of a Defense IV type.

In contrast, another genotype comparison showed that Fe exclusion can also lead to tolerance. Comparing Fe-sensitive and Fe-tolerant genotypes that were typically cultivated in southern Brazil, authors observed very distinct transcriptional responses to Fe excess in roots (Stein et al., 2019). While the sensitive genotype responded as expected, decreasing the expression of Fe uptake genes, the tolerant one upregulated cell wall biosynthesis and lignification genes. Interestingly, the tolerant genotype was able to avoid Fe accumulation in both roots and shoots, suggesting that cell wall modifications upon Fe excess can decrease Fe uptake. Such Fe exclusion mechanisms were further supported by a clear increase in ectopic root lignin deposition close to exodermis and endodermis, which could restrict Fe radial transport, as observed in *A. thaliana* (Reyt et al., 2021). Therefore, this work suggested that Fe exclusion by cell wall modifications could be a mechanism for Fe tolerance (Stein et al., 2019).

Analysis of 16 lowland rice varieties found two clearly contrasting cultivars of Fe excess tolerance, Lachit (Fe-tolerant cultivar) and Hacha (Fe-sensitive cultivar) (Kar et al., 2021). Lachit accumulated less Fe in leaves compared to Hacha, pointing to a shoot exclusion mechanism. Hacha showed higher Fe accumulation in shoots, leading to increased lipid peroxidation and leaf rolling. Transcriptomic analyses suggested that the sensitive genotype undergoes larger ferroptosis, while it also had more Gene Ontology terms associated with oxidative stress. The Fe-tolerant genotype (Lachit) also showed higher gene expression of *OsFER2*, *OsNodulin-like2*, and *OsNRAMP4* and lower expression of *OsNRAMP1*, *OsNAAT1*, *OsHMA2*, *OsHMA9*, and *OsTOM2*, as well as *OsYSL2* and its regulator *OsIDEF2*, compared to the Fe-sensitive one (Hacha) (Kar et al., 2021). Altogether, the results suggest that the tolerance genotype is likely to reduce Fe uptake as well as restrict Fe root-to-shoot translocation (Kar et al., 2021).

## 7 Iron excess tolerance in rice wild relatives

Asian rice *Oryza sativa* L., which was domesticated from *Oryza rufipogon* and *Oryza nivara*, is the most consumed rice species (Huang et al., 2012; Stein et al., 2018). The African rice *Oryza glaberrima* was independently domesticated from another wild progenitor, *Oryza barthii* (Wang et al., 2014), and is a staple food in West Africa. In addition to these two cultivated species, the *Oryza* genus has another 22 species, which altogether are excellent sources for gene and allele discovery and can contribute with a variety of traits to improve domesticated rice (Ricachenevsky and Sperotto, 2016; Menguer et al., 2017; Bierschenk et al., 2020; de Oliveira et al., 2020; Wairich et al., 2021). The evolutionary history of the *Oryza* genus spans approximately 15 million years, a period that has allowed for diversity among species (Vaughan et al., 2003; Menguer et al., 2017). A total of 11 different genome types occur in this genus, with a variation of up to 3.6 times in size (Jacquemin et al., 2013; Menguer et al., 2017; Stein et al., 2018). The genomic types are diploid n =12 (AA, BB, CC, EE, FF, and GG) and allotetraploid n = 24 (BBCC, CCDD, KKLL, HHJJ, and HHKK) (Chen et al., 2019).

Although some successful cases of introgression have already been reported (Menguer et al., 2017), the potential use of such wild relatives is still untapped. To overcome limitations, several species from the *Oryza* genus, particularly the ones from the *O. sativa* complex (i.e., diploid, closely related to *O. sativa* and having an AA genome) already have reference genomes sequenced, and resources such as introgression lines, and seed banks for trait and gene discovery, are available (Arbelaez et al., 2015; Menguer et al., 2017; Stein et al., 2018; Wairich et al., 2021; Zhang et al., 2022).

In the first effort to identify Fe tolerance trait among *Oryza* genus species, 75 accessions from 21 species were screened, including 58 wild relatives and 16 domesticated species (Bierschenk et al., 2020). The authors tested both acute and chronic Fe excess stress. In acute stress, *Oryza* wild accessions were both among the most sensitive and the most tolerant, with two genotypes from *Oryza meridionalis* ranking among the most tolerant to Fe excess. In chronic stress, *O. sativa* genotypes were the most tolerant, although the two *O. meridionalis* were also tolerant. Still, *O. sativa* genotypes were also among the most tolerant genotypes in both treatments, indicating that wide variation in Fe tolerance is found in both domesticated and wild species. As authors found differences in Fe accumulation, the work also suggested that in wild species, distinct mechanisms may be found (Bierschenk et al., 2020). Importantly, *O. meridionalis* was suggested as a possible donor of tolerance traits.

Interestingly, analyses of introgression lines of *O. meridionalis* into *O. sativa* (Arbelaez et al., 2015) resulted in the identification of one introgression line with shoot-based tolerance (Wairich et al., 2021). *O. meridionalis* is endemic to Northern Australia, growing in temporary lagoons and swamps with Fe-rich soils (Cogle et al., 2011), and is part of the AA genome *Oryza* complex. *O. meridionalis* was shown to accumulate similar Fe concentrations in roots but lower concentrations in aerial tissues, suggesting that it excludes Fe from shoots but retains it in roots (Defense II) (Wairich et al., 2021). Interestingly, the most Fe-tolerant introgression line showed a distinct mechanism, with a shoot-based mechanism in which plants were able to sequester Fe in the leaf sheath while avoiding Fe accumulation in the photosynthetically most active tissue, the leaf blade. Transcriptional analyses suggested several candidate genes that are located in the introgression region, although no clear candidate gene was found yet (Wairich et al., 2021). Still, the work showed that exploring rice wild relatives using introgression lines may result in new mechanisms for Fe toxicity tolerance. The identification of the mechanism of Fe tolerance in an introgression line genotype resulting from a cross between one Fe-sensitive species and a Defense II species demonstrates that *O. meridionalis* may have other mechanisms for Fe excess tolerance that are not observed when Fe exclusion from shoots is employed.

## 8 Genes encoding proteins involved in Fe excess response in rice

In comparison to Fe deficiency response, Fe excess response in rice is less well understood mechanistically. Few characterized genes were linked to Fe overload responses, which may be derived from the fact that Fe excess is a particular problem for rice that is not shared with *A. thaliana* and other model species. Grass species such as *Paspalum urvillei*, *Setaria parviflora*, and *Imperata cylindrica* show tolerance to Fe excess and may even hyperaccumulate it (Fuente et al., 2016; de Oliveira et al., 2020). Still, work on rice is uncovering how this crop can respond to Fe overload and which genes are important in dealing with Fe stress.

Ferritins are proteins that form nanocages made of 24 subunits and store several Fe atoms in the central cavity (Briat et al., 2010). However, these proteins are also associated with processes related to plant development and resistance to damage due to oxidative stress. In some species, ferritin genes are associated with higher tolerance to biotic (Deák et al., 1999; Garcia-Mata et al., 2001; Dellagi et al., 2005; Nguyen et al., 2022) and abiotic stresses, such as drought and salinity (Zhang et al., 2023). In *A. thaliana*, there are four ferritin genes, *AtFER1* to *AtFER4*, which were characterized and shown to encode proteins involved in protecting plants from Fe excess-induced oxidative stress (Ravet et al., 2009; Reyt et al., 2015). The four proteins are localized to the chloroplast, while AtFER4 is dual-targeted to chloroplasts and mitochondria (Zancani et al., 2004; Ravet et al., 2009). In rice, however, there are two ferritin genes, *OsFER1* and *OsFER2* (Gross et al., 2003). Based mainly on expression patterns, the proposed roles of these two proteins involve defense against Fe-inducing oxidative stress (Silveira et al., 2009; Stein et al., 2009; Quinet et al., 2012; Wu et al., 2017; Aung et al., 2018b; Stein et al., 2019; Kar et al., 2021; Wairich et al., 2021; Regon et al., 2022). *OsFER2* also increases expression when rice plants are exposed to Paraquat, copper, and sodium nitroprusside (Stein et al., 2009). In contrast, the downregulation of expression of *OsFER2* in plants submitted to sulfate deficiency was also observed, suggesting a cross-talk between sulfate and Fe metabolism, as well as regulation of ferritin genes by sulfur nutrition in rice (Ohkama-Ohtsu et al., 2004). Recently, *OsFER2* was shown to positively regulate ferroptotic cell death in rice plants infected with *M. oryzae* (Nguyen et al., 2022), suggesting an important cross-talk between Fe homeostasis and biotic stress.

The Ferric Reductase Oxidase (FRO) proteins are associated with Fe uptake in roots, reducing Fe(III) to Fe(II) under Fe deficiency, making Fe(II) readily available for Fe(II) transporters such as IRT1 (Brumbarova et al., 2015). However, grasses do not use membrane-bound reductases for Fe uptake, and until recently, the role of FROs in this group of plants was not yet described. While *A. thaliana* has eight FRO genes in its genome, rice has only two. *OsFRO1* is clustered with *AtFRO7*, which was characterized as a reductase bound to the chloroplast envelope, involved in Fe reduction prior to chloroplast uptake (Jeong et al., 2008). The second gene, *OsFRO2*, is truncated, lacking domains found in other FROs (Li et al., 2019), suggesting that it could be a pseudogene. *OsFRO1* was shown to be expressed mainly in leaves and to be downregulated upon Fe excess (Li et al., 2019). OsFRO1 protein was also shown to be localized to the vacuole. Plants silenced for *OsFRO1* showed increased Fe excess tolerance, while plants overexpressing *OsFRO1* showed slightly higher sensitivity. Silencing *OsFRO1* also decreased Fe concentration in shoots and decreased leaf ROS formation. The data suggest a role for *OsFRO1* in vacuolar Fe reduction, which would make it more available for the cytoplasm (Li et al., 2019). In agreement with that, a forward screening using 4,500 fast-neutron mutagenized rice lines found that lines tolerant to Fe excess were mutated for *OsFRO1* as well (Ruengphayak et al., 2015), establishing *OsFRO1* as an important gene involved in Fe homeostasis and tolerance. Still, the precise role of the protein is unclear, as reductase activity was not detected (likely due to the vacuolar localization, making it harder to detect compared to plasma membrane bound reductases; Li et al., 2019).

*OsAKT1* (potassium ion channel gene) encodes an inward potassium ion channel that localizes to the plasma membrane and plays a critical role in potassium uptake in rice roots (Li et al., 2014). A genome-wide association study has linked *OsAKT1* to genotypic differences related to the shoot Fe concentration (Matthus et al., 2015). Knockout lines for *OsAKT1* were more sensitive to Fe toxicity, showing increased leaf bronzing symptoms, H_2_O_2_ detection in leaves, and higher levels of Fe translocation to the xylem and reduced photosynthesis efficiency, compared to null-segregating wild type. These results suggest that *OsAKT1* may play a role in Fe toxicity tolerance in rice, which could involve potassium homeostasis affecting Fe translocation from root-to-shoot tissue (Wu et al., 2019).

*OsNAS3* is a gene involved in nicotianamine synthesis (Higuchi et al., 2001). In rice, there are three NAS genes, and while *OsNAS1* and *OsNAS2* are induced under Fe deficiency and repressed by Fe excess, *OsNAS3* shows the opposite expression pattern, being induced by Fe excess in roots, DC, stems, and old and new leaves (Aung et al., 2018b; Wairich et al., 2019). The *osnas3* knockout plants showed growth defects compared to wild type (WT) when cultivated under Fe excess conditions. In addition, higher leaf bronzing symptoms and higher Fe accumulation in the shoot were observed in knockout plants when compared to WT plants due to enhanced Fe translocation from roots to shoots, reinforcing the contribution of *OsNAS3* to Fe detoxification in rice (Aung et al., 2019).

The vacuolar iron transporter (VIT) genes have the potential to contribute to Fe excess response since VIT proteins are able to transport Fe into vacuoles. The first protein characterized in the family was *A. thaliana* AtVIT1, which has a role in the proper localization of Fe in the seeds (Kim et al., 2006). However, AtVIT1 does not seem to have a function in protecting *A. thaliana* plants from high Fe concentrations. In rice, there are two VIT proteins, namely, OsVIT1 and OsVIT2. Mutations in *OsVIT2* and *OsVIT1* result in increased Fe accumulation in seeds, making this gene family a promising candidate for biofortification (Zhang et al., 2012b; Bashir et al., 2013; Che et al., 2021; Kandwal et al., 2022; Wairich et al., 2022). *OsVIT2* is consistently upregulated when plants are exposed to high Fe (**Table 2**; Zhang et al., 2012b). Considering that rice plants are prone to Fe overload, a possible role for VIT transporters in Fe detoxification is possible. However, single OsVIT1 and OsVIT2 mutants showed no difference in Fe excess sensitivity (Zhang et al., 2012b; Che et al., 2021). Therefore, it is possible that OsVIT1 and OsVIT2 play partially redundant roles under Fe excess.

Early work suggested that OsWRKY80 was a possible Fe excess-induced transcription factor (Ricachenevsky et al., 2010). Later work suggested that other WRKY transcription factors could be involved in Fe excess regulation (Aung and Masuda, 2020). Recent efforts in using machine learning uncovered several potential *cis*-elements that could regulate Fe excess-responsive genes, as well as candidate genes involved in their transcriptional control (Kakei et al., 2021).

At the post-transcriptional level, rice HRZ (Hemerythrin motif-containing Really interesting new gene- and Zinc-finger) proteins OsHRZ1 and OsHRZ2 are E3 ubiquitin ligases that can bind Fe and function as Fe sensors. Both proteins downregulated key Fe uptake proteins by proteolysis, therefore functioning as negative feedback to limit the Fe deficiency response (Kobayashi et al., 2013). Interestingly, OsHRZ1 and OsHRZ2 were also shown to be important for protecting rice plants from excessive Fe uptake, presumably by sensing Fe and decreasing Fe uptake when supply is available (Aung et al., 2018a). Once the plant receives an Fe excess signal, Defense I may work to inhibit root Fe uptake by the suppression of Fe uptake- and transport-related genes where HRZs are crucial to suppressing these genes (Aung and Masuda, 2020). Taken together, these data provide information on how to control Fe excess responses, an important step toward developing Fe-tolerant rice genotypes.

## 9 Conclusion

Rice is a major staple food for humans. Fe excess affects waterlogged rice worldwide, decreasing productivity and affecting farmers and consumers. There are proportionally more studies on the Fe deficiency responses of this crop. This is likely a consequence of Fe deficiency occurrence in several other plants in field conditions and of a large body of accumulated knowledge in the model species *A. thaliana* (Brumbarova et al., 2015), which aids comparisons for Fe deficiency studies as well as other aspects of Fe homeostasis in rice (Kobayashi and Nishizawa, 2012; Ricachenevsky et al., 2018; Whitt et al., 2020). However, understanding Fe excess responses and the genetic bases of Fe excess tolerance in rice would have a major impact and should be a focus of the community. We propose the following three key points that should be addressed in future research: 1) cloning causative genes associated with QTL for Fe excess tolerance, within rice natural variation as well as among wild rice species; 2) comprehensively characterizing genes encoding proteins that may be important for Fe excess responses, such as those frequently identified as upregulated in multiple papers; and 3) combining genes that may be overexpressed, expressed under control of specific promoters, or edited using CRISPR-Cas9 to generate Fe excess-tolerant genotypes that can withstand stress in field conditions. With that, we should be able to provide solutions to farmers where Fe excess is affecting rice production.

## Author contributions

AW: Conceptualization, Writing – original draft, Writing – review & editing. MSA: Conceptualization, Writing – original draft, Writing – review & editing. FR: Conceptualization, Funding acquisition, Project administration, Supervision, Writing – original draft, Writing – review & editing. HM: Conceptualization, Funding acquisition, Project administration, Supervision, Writing – original draft, Writing – review & editing.
